# Comparative Evaluation of Background Subtraction Algorithms in Remote Scene Videos Captured by MWIR Sensors

**DOI:** 10.3390/s17091945

**Published:** 2017-08-24

**Authors:** Guangle Yao, Tao Lei, Jiandan Zhong, Ping Jiang, Wenwu Jia

**Affiliations:** 1 Institute of Optics and Electronics, Chinese Academy of Sciences, P.O. Box 350, Shuangliu, Chengdu 610209, China; taoleiyan@ioe.ac.cn (T.L.); zjdwell@163.com (J.Z.); jiangping@ioe.ac.cn (P.J.); 2 School of Optoelectronic Information, University of Electronic Science and Technology of China, No. 4, Section 2, North Jianshe Road, Chengdu 610054, China; 3 University of Chinese Academy of Sciences, 19 A Yuquan Rd, Shijingshan District, Beijing 100039, China; 4 China Huayin Ordnance Test Center, Huayin 714200, China; shrek2012@163.com

**Keywords:** background subtraction, remote scene, IR video sequence, MWIR sensor, background modeling, foreground detection

## Abstract

Background subtraction (BS) is one of the most commonly encountered tasks in video analysis and tracking systems. It distinguishes the foreground (moving objects) from the video sequences captured by static imaging sensors. Background subtraction in remote scene infrared (IR) video is important and common to lots of fields. This paper provides a Remote Scene IR Dataset captured by our designed medium-wave infrared (MWIR) sensor. Each video sequence in this dataset is identified with specific BS challenges and the pixel-wise ground truth of foreground (FG) for each frame is also provided. A series of experiments were conducted to evaluate BS algorithms on this proposed dataset. The overall performance of BS algorithms and the processor/memory requirements were compared. Proper evaluation metrics or criteria were employed to evaluate the capability of each BS algorithm to handle different kinds of BS challenges represented in this dataset. The results and conclusions in this paper provide valid references to develop new BS algorithm for remote scene IR video sequence, and some of them are not only limited to remote scene or IR video sequence but also generic for background subtraction. The Remote Scene IR dataset and the foreground masks detected by each evaluated BS algorithm are available online: https://github.com/JerryYaoGl/BSEvaluationRemoteSceneIR.

## 1. Introduction

Background subtraction is a common way to detect and locate moving objects in video sequences. It is the first step for all kinds of applications in the computer vision field, such as video analysis, object tracking, video surveillance, object counting, traffic analysis, etc. BS is related to the following problems: background modeling, foreground extraction, change detection, foreground detection and motion detection.

Since the 1990s a large number of BS algorithms have been proposed. Also different kinds of BS datasets and benchmarks have been released to evaluate BS algorithms. Many reviews and evaluation papers have been published to-date. In this paper, a Remote Scene IR Dataset is provided which is captured by our designed medium-wave infrared sensor. This dataset is composed of 1263 frames in 12 video sequences representing different kinds of BS challenges and it is annotated with pixel-wise foreground ground truth. We firstly selected 16 important and influential BS algorithms, and conducted a serious of comprehensive experiments on this Remote Scene IR Dataset to evaluate the performance of these BS algorithms. We also conducted an overall experiment on the 24 BS algorithms from the BGSLibrary [1] which is a powerful BS library. The results and conclusions in this paper provide valid references to develop new BS algorithm for remote scene IR video sequences, and some of them are not only limited to remote scenes or IR video sequences, but also generic for background subtraction, such as experimental results concerning ghosts, high and low foreground movement speeds, memory and processor requirements, etc.

### 1.1. Motivation and Contribution

Although numerous review and evaluation on background subtraction have been published in the literature, there are still several reasons that motivated this evaluation paper:

(1) The released BS datasets [2,3,4,5] do not focus on the remote scene. Background subtraction and moving targets detection in remote scene video is important and common to lots of fields, such as battlefield monitoring, intrusion detection and outdoor remote surveillance. Remote scene IR video sequences present typical characteristics: small and even dim foreground, less color, texture and gradient information in the foreground (FG) and background (BG), which causes difficulty for BS and affects the performance of BS. It is necessary to develop a remote scene IR dataset and evaluate BS algorithms on it.
(2) The challenges of high and low speeds of foreground movement have been identified in previous works [6,7], and are presented in the released cVSG dataset [6]. For the challenge of high speed of foreground movement, if the speed is high enough, such as beyond 1 self-size per frame, which means that there is no overlap between the foregrounds in two sequential frames, some BS algorithms would yield hangover as shown in Figure 14. In the BS paradigm, each pixel is labeled as foreground or background. For the challenge of low speed of foreground movement, if the speed is low enough, especially below 1 pixel per frame, it is much more difficult to distinguish the foreground pixels. It is important to evaluate BS algorithms to cope with these two challenges. The speed units self-size/frame and pixel/frame are adopted respectively for the high and low speed challenges in this evaluation paper.
(3) In the published evaluation papers, there is not enough experimental data and analysis on some identified BS challenges. Camouflage is an identified challenge [3,4,8,9] which is caused by foreground that has similar color and texture as the background, but these papers do not provide a video sequence representing it. Reference [2] provided a synthetic video sequence representing camouflage challenges concerning color. Camouflaged foreground is unavoidable in video surveillance. It is important to conduct evaluation experiments on real video sequences representing this challenge.
(4) It is illogical to evaluate the capability of BS to handle kinds of challenges based on the whole video sequence or category with same evaluation metrics. Previous works [2,3,4] always group the video sequences into several categories according to the type of challenge, and evaluate the capability of BS algorithms to handle these challenges with same evaluation metrics based on the whole category. Actually some challenges such as camera jitter only last for several frames or impact several frames. Some challenges such as shadow and ghosting only occupy small parts of the frame. To evaluate the capability of BS to handle these challenges, it is logical to evaluate the performance change caused by these challenges with proper evaluation metrics or criteria. As examples, for camera jitter, we should focus on the frames after it occurs and the changes of performance; for ghosting, we should focus on whether it appears and how many frames it lasts for; for high speed of foreground movement, we should focus on whether the hangover phenomenon appears and how many frames the hangover lasts for.
(5) There is no detailed implementation and parameter setting in some BS algorithm papers [10,11] and previous evaluation papers [5,8,12]. Because of the different implementations, the same BS algorithm often performs differently. It is reasonable to detail the implementation and parameter setting of the evaluated BS algorithms.
(6) The comparison is not fair in some previous evaluation experiments. Post-processing is a common way to improve the performance of BS. BS algorithms [13,14,15,16,17] utilize and benefit from post-processing as part of the BS process. It would be fairer to remove post-processing from these BS algorithms and evaluate the BS algorithms without and with post-processing, respectively.

The contributions of this paper can be summarized as follows:

(1) A remote scene IR BS dataset captured by our designed MWIR sensor is provided with identified challenges and pixel-wise ground truth of foreground.
(2) BS algorithms are summarized in six important issues which are used to describe the implementation of BS algorithms. The implementations of the evaluated BS algorithms are detailed according to these issues. The parameter settings are also presented in this paper.
(3) We improved the rank-orders used in the CVRP CDW challenge [3,4] by combining several evaluation metrics.
(4) BS algorithm evaluation experiments were conducted on the proposed remote scene IR dataset. The overall performance of the evaluated BS algorithms and processor/memory requirements are compared. Proper evaluation metrics and criteria are selected to evaluate the capability of BS to handle the identified BS challenges represented in the proposed dataset.

### 1.2. Organization of This Paper

The rest of this paper is organized as follows: in Section 2, previous related works are reviewed, including previous BS datasets and evaluation papers. In Section 3, an overview of the BS algorithm and new mechanisms of BS are presented. Section 4 introduces the designed MWIR sensor, the proposed Remote Scene IR BS Dataset and the challenges represented in each video sequence. Section 5 details the setup of evaluation experiments, evaluation metrics and rank-order rules. In Section 6 we discuss the experimental results, and compare the overall performance of the evaluated BS algorithms and their capability to handle the identified challenges. We also compare their processor/memory requirements. In Section 7, conclusions and future work perspectives are presented.

## 2. Previous Works

### 2.1. Previous Datasets

In the past, numerous datasets and benchmarks have been released to evaluate BS algorithms. The early datasets (IBM [18], Wallflower [19], PETS [20], CMU [21], ViSOR [22] etc.) were developed for tracking methods, and only part of these datasets provided bounding box ground truths. Some of these early datasets are not identified with the challenges of BS. Recently, new datasets were developed to evaluate BS algorithms, which provide the pixel-wise ground truth of foreground, even pixel-wise shadow and Region of Interest (ROI). The specific BS challenges are identified in these datasets. Table 1 introduces the datasets developed recently.

The Stuttgart Artificial Background Subtraction (SABS) dataset is a synthetic dataset which consists of video sequences representing nine different background subtraction challenges for outdoor video surveillance [2].

The Change Detection Workshop 2012 (CDW2012) [3] dataset was developed for the CVPR2012 Change Detection Workshop challenge. It consists of 31 realistic videos spanning six categories: Baseline, Dynamic Background, Camera Jitter, Intermittent Object Motion, Shadows and Thermal.

The Change Detection Workshop 2014 (CDW2014) [4] dataset was developed for the CVPR2014 Change Detection Workshop challenge. It extends the CDW2012 dataset with a new set of realistic videos representing four additional categories: Challenging Weather, Low Frame-Rate, Night, PTZ and Air Turbulence.

The Background Modeling Challenge (BMC) [5] dataset was developed for the comparison workshop of BS algorithms in ACCV2012. It is composed of 20 synthetic videos and nine realistic videos. Part of the videos are labeled with pixel-wise ground truth of foreground.

The Maritime Detection, Classification, and Tracking (MarDCT) [23] dataset consists of videos and images from multiple sources and different scenarios. The aim of this dataset is to provide a set of videos that can be used to develop intelligent surveillance systems for the maritime environment.

The Camplani, Blanco, Salgado (CBS) [24,25] RGB-D dataset provides five sequences of indoor environments captured by a Microsoft Kinect RGB-D camera. Each sequence represents different identified challenges.

The Fernandez-Sanchez, Diaz, Ros (FDR) [26,27] RGB-D dataset contains two different sets of sequences: one (four video sequences) was recorded by a stereo camera combined with three disparity estimation algorithms; the other (four video sequences) was recorded by a Microsoft Kinect RGB-D camera.

### 2.2. Previous Evaluation and Review Papers

A number of evaluations and reviews about BS can be found in the literature published to date. The early papers [28,29,30,31,32,33,34,35,36,37,38,39] did not evaluate or review the new BS algorithms. Some of these papers conducted evaluation experiments on their own, used non-public datasets, and some of these papers did not evaluate BS algorithms for the identified challenges. Papers [40,41] only evaluated statistical BS algorithms.

Since 2010, some new papers were published which evaluated and reviewed BS algorithms on public datasets with identified challenges. The important evaluation and review papers are introduced in Table 2.

Brutzer et al. [2] firstly identified the main challenges of background subtraction, and then compared the performance of nine background subtraction algorithms with post-processing and their capability to handle these challenges. This paper also introduced a new evaluation dataset with accurate ground truth annotations and shadow masks which enables precise in-depth evaluation of the strengths and drawbacks of BS algorithms.

Goyette et al. [3] presented various aspects of the CDW2012 dataset used in the CVPR2012 CDW Challenge. This paper also discussed quantitative performance metrics and comparative results for over 18 BS algorithms.

Wang et al. [4] presented the CDW2014 datasets used in the CVPR2014 CDW Challenge, and described every category of dataset that incorporates challenges encountered in BS. This paper also provided an overview of the results of more than 16 BS algorithms.

Vacavant et al. [5] presented the BMC dataset with both synthetic and real videos and evaluated six BS algorithms on this dataset. The BMC dataset focuses on outdoor scenes with weather variations such as wind, sun or rain. This paper also proposed some evaluation criteria and a free software to compute them.

Sobral et al. [42] compared the 29 BS algorithms on the BMC dataset, and conducted experimental analysis to evaluate robustness of BS algorithms and their practical performance in terms of computational load and memory usage.

Dhome et al. [12] proposed a BS algorithm evaluation dataset developed by LIVIC SIVIC simulator [43], and conducted evaluation of six BS algorithms on this dataset based on several evaluation metrics.

Benezeth et al. [8] presented a comparative study of seven BS algorithms on various synthetic and realistic video sequences representing kinds of challenges. These sequences are a collection from other BS datasets.

Bouwmans [9] provided a complete survey of the traditional and recent approaches. First, this paper categorized BS algorithms found in the literature and discussed them. Then this paper presented the available resources, datasets and libraries. Finally, several promising directions for future research were suggested, but there were no evaluation experiments for BS algorithms.

## 3. Overview of Background Subtraction

### 3.1. Description of Background Subtraction Algorithm

**Figure 1:**
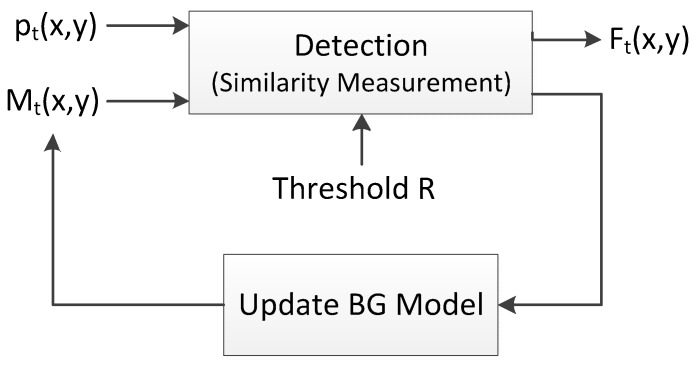
Paradigm of Background Subtraction.

Many BS algorithms have been designed to segment the foreground objects from the background of a sequence, and generally share the same scheme [42], which is shown in Figure 1. A background (BG) model $M_{t}\left(x,\;y\right)$ is constructed and maintained for pixel $p_{t}\left(x,\;y\right)$ at time t. If $p_{t}\left(x,\;y\right)$ is similar with its background model $M_{t}\left(x,\;y\right)$, it is labeled as a background pixel or it is a foreground pixel. We summarize six important issues of BS which are used to describe the implementation of BS algorithms. Initiation, detection and updating are the steps of background subtraction as mentioned in [9,42,44].

(1) Features: What features are selected for each pixel?

**Figure 2:**
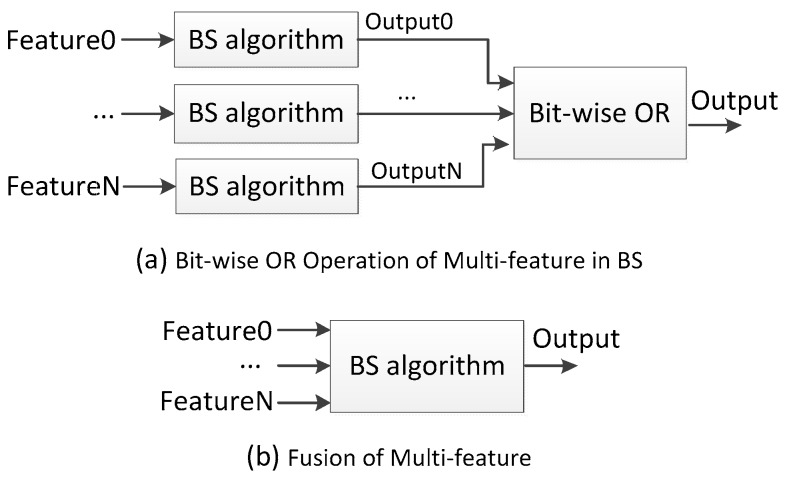
Multi-feature fusion and bit-wise OR operation in background subtraction.

Pixel colors including RGB color, YUV color and HSV color, etc., are the features commonly used in BS. Co-occurrence, chromaticity and gradient features are also employed in BS algorithms. Recently different kinds of texture features are also employed. References [45,46,47] adopt Local Binary Pattern (LBP) and modified LBP texture features; references [48,49,50] adopt Local Binary Similarity Pattern (LBSP) texture features. To capture much more information, some BS algorithms adopt multi-features with bit-wise OR operation or fusion. Bit-wise OR operation of multi-features is illustrated in Figure 2a. Pixels are distinguished using each feature independently, and the final result comes from a bit-wise OR operation. Reference [51] applies chromaticity and gradient features with bit-wise OR operations. Fusion of multi-features as illustrated in Figure 2b is much more common. Pixels are distinguished using the combined multi-features, and each feature plays its own role and makes different contributions, and these features are even assigned weights. Reference [52] measures the similarity between pixels and its BG model using weighted features: RGB color and gradient. Reference [53] utilizes fuzzy integrals to fuse the Ohta color and gradient for background model. Reference [54] computes the Gaussian mixture density for each pixel with RGB color, gradient and haar-like features.

(2) BG Model: What variance parameters of features are saved in the background model?

Besides the original value of the selected features, BS algorithms also save variance parameters of features in the BG model, such as average, median, density, neuronal map, dictionary, etc. Reference [55] saves a buffer of color values over time in the BG model to get the median of them. References [10] and [56] respectively save the running median and running average of color in the BG model. Reference [57] saves a temporal standard deviation computed by a Sigma-Delta filter. References [58,59,60] save a history of color in BG model. Reference [52] saves a history of color and gradient in BG model. References [61,62] save the density in BG model. Reference [11] saves statistics (mean and covariance) of features. References [14,15,63] save several statistics of features with weights in BG model. References [17,64] use an artificial neural map as BG model.

(3) Initialization: How to initialize a BG model?

Initialization is the first step of background subtraction. A BG model is initialized using the frames at the beginning of the video sequence. References [11,59,60] initialize the BG model using only one frame. References [17,52] initialize the BG model using several frames and detect the foreground on initialization, while [13,61] also initialize BG model using several frames but there is no foreground detection in initialization.

(4) Detection: How to measure the similarity between pixels and the background model?

Detection is the second step of background subtraction, which is also referred to as segmentation. In this step, the similarity between a pixel and its BG model is measured to label the pixel as background or foreground. As illustrated by Equation (1), if the similarity is beyond some threshold R, the pixel is labeled as background, otherwise it is labeled as foreground. To measure the similarity, references [10,57,59] apply L1 distance, while [11,17] apply L2 distance, [16,61] apply probability and [45] applies histogram intersection:

(1) $$F_{t}(x,y)=\left\{ \begin{array}{l} 1\;\mathrm{dist}(p_{t}(x,y),\;M_{t}(x,y))\;>R\\ 0\;\mathrm{otherwise} \end{array}\right.$$

(5) Update: How to update BG model?

BG model update is the last step of background subtraction, which is also referred to as BG model maintenance. If a pixel is labeled as background, its BG model should be updated. There are six update strategies: non-update, iterative update, first-in-first-out (FIFO) update, selective update, random update and hybrid update. In a static frame difference algorithm, a static frame is set manually as the BG model, so there is no update. References [11] iteratively update the BG model with an IIR filter, which is illustrated in Equation (2). The learning rate α is a constant in [0, 1], which determines the speed of the adaptation to the scene changes:

(2) $$M_{t+1}(x,y)=(1-\alpha )M_{t}(x,y)+\alpha p_{t}(x,y)$$

References [58,61] apply a FIFO update strategy. References [65,66] selectively replace the codeword in the BG model. References [52,59,60] adopt a random replace strategy. References [13,16,45] use the hybrid update in which more than one update strategies is adopted. Reference [13] removes the features with minimum weight and iteratively updates the BG model with new features. Reference [16] adopts iterative and selective updates, respectively, for gradual background change and “once-off” background change. In [45], if measured proximity is below a threshold for all feature histograms, a selective update strategy is adopted, or an iterative update is adopted.

(6) Multi-Channel: How to conduct background subtraction in multi-channel video sequence?

**Figure 3:**
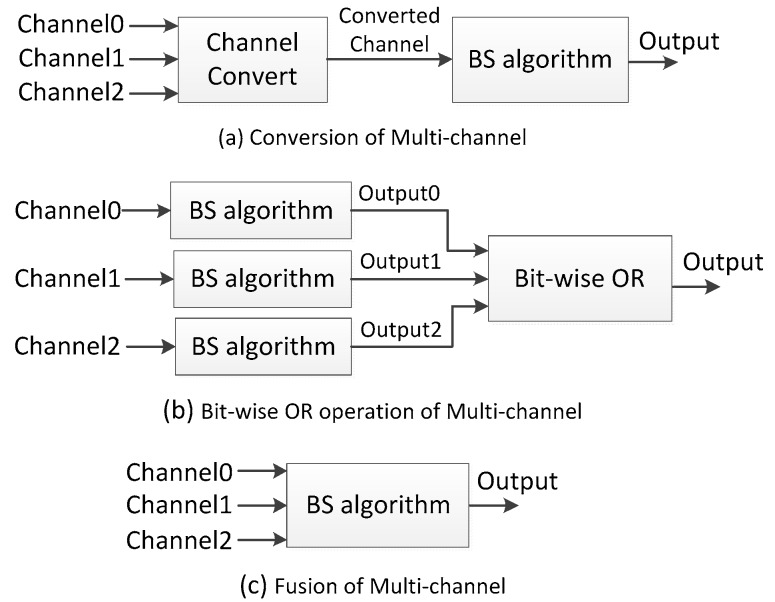
Multi-channel processing in background subtraction.

For multi-channel video sequences, there are three processing schemes: conversion, bit-wise OR and fusion, which are shown in Figure 3.

Reference [46] and Gray-ViBe in [59] first convert the color frames to gray frames, and then conduct background subtraction on the gray frames. Reference [52] runs background subtraction in each channel independently, and the final result comes from a bit-wise OR operation. Many more BS algorithms [13,14,61] employ multi-channel fusion methods which processes BS in a multi-channel space.

### 3.2. New Mechanisms in BS Algorithm

**Figure 4:**
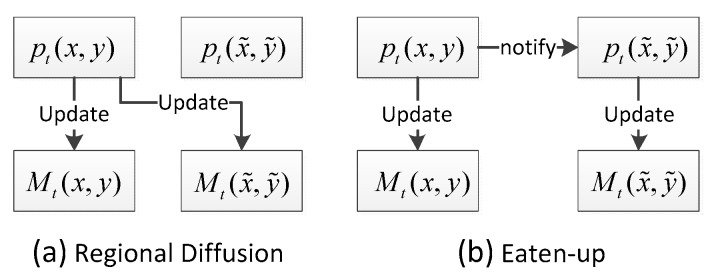
Regional diffusion and eaten-up in BG model update.

Recent BS algorithms employ some new technologies and ideas to improve the performance, such as regional diffusion, eaten-up and feedback. Regional diffusion of background information proposed in [59,60] is used to update BG model, which is also referred to as spatial diffusion or spatial propagation. Given a pixel $p_{t}\left(x,\;y\right)$ with BG model $M_{t}\left(x,\;y\right)$ and its neighborhood $p_{t}\left(\tilde{x},\;\tilde{y}\right)$ with BG model $M_{t}\left(\tilde{x},\;\tilde{y}\right)$, if $p_{t}\left(x,\;y\right)$ is labeled as background, not only $M_{t}\left(x,\;y\right)$ but also $M_{t}\left(\tilde{x},\;\tilde{y}\right)$ is updated using the feature of $p_{t}\left(x,\;y\right)$. Figure 4a illustrates how the regional diffusion works in BG model update. This mechanism propagates background pixels spatially, which ensures spatial consistency. The advantage of regional diffusion is that ghost will be slowly included into the background, and BS is robust to camera jitter.

Eaten-up proposed in [52] is also used to update BG model. Different from regional diffusion, the eaten-up mechanism is that if pixel $p_{t}\left(x,\;y\right)$ is label as background, $M_{t}\left(\tilde{x},\;\tilde{y}\right)$ is updated with the features of $p_{t}\left(\tilde{x},\;\tilde{y}\right)$, not the features of $p_{t}\left(x,\;y\right)$. Figure 4b illustrates how the eaten-up method works in BG model update. In this mechanism, a neighboring pixel, which might be foreground, can be updated as well. This means that certain foreground pixels at the boundary will gradually be included into the background. The advantage of eaten-up is that erroneous foreground pixels will quickly vanish.

**Figure 5:**
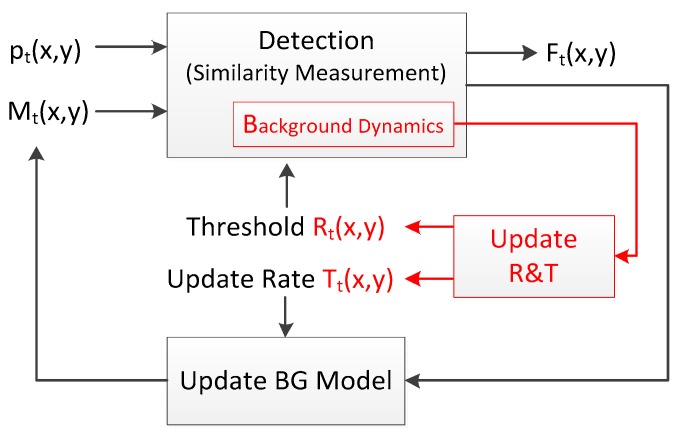
Feedback loop used in PBAS.

Feedback loop is the key of the adaptive BS algorithm. It is used to dynamically adjust the parameters of BS. Reference [52] applies feedback loops based on background dynamics to dynamically adjust the decision threshold and learning rate. In [50,67], feedback loops based on temporal smoothing are used to dynamically adjust the feature-space distance threshold, persistence threshold and update rate. In almost the same way [50,67], [68] apply feedback loops to dynamically adjust the feature-space distance threshold and update rate. In Figure 5, an overview of PBAS is shown [52].

Compared with Figure 1, there is an additional feedback loop. This feedback loop steered by the background dynamic is used to adaptively adjust the parameters at runtime for each pixel separately.

## 4. MWIR Sensor and Remote Scene IR Dataset

**Figure 6:**
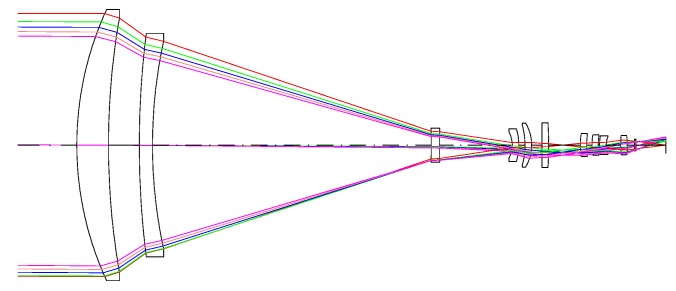
Schematic of the medium-wave infrared imaging sensor.

In this evaluation paper, the Remote Scene IR Dataset is proposed. All the video sequences in this dataset were captured by our designed medium-wave infrared sensor. Figure 6 is the schematic of this medium-wave infrared imaging sensor. This sensor applies a highly sensitive thermoelectrically cooled mercury cadmium telluride (MCT) detector which adapts to dark, smoke and strong illumination because of its transmittance ability, and can be used to detect and track objects in remote scenes. The key optical, electrical, physical specifications of this MWIR sensor are presented in Table 3.

**Figure 7:**
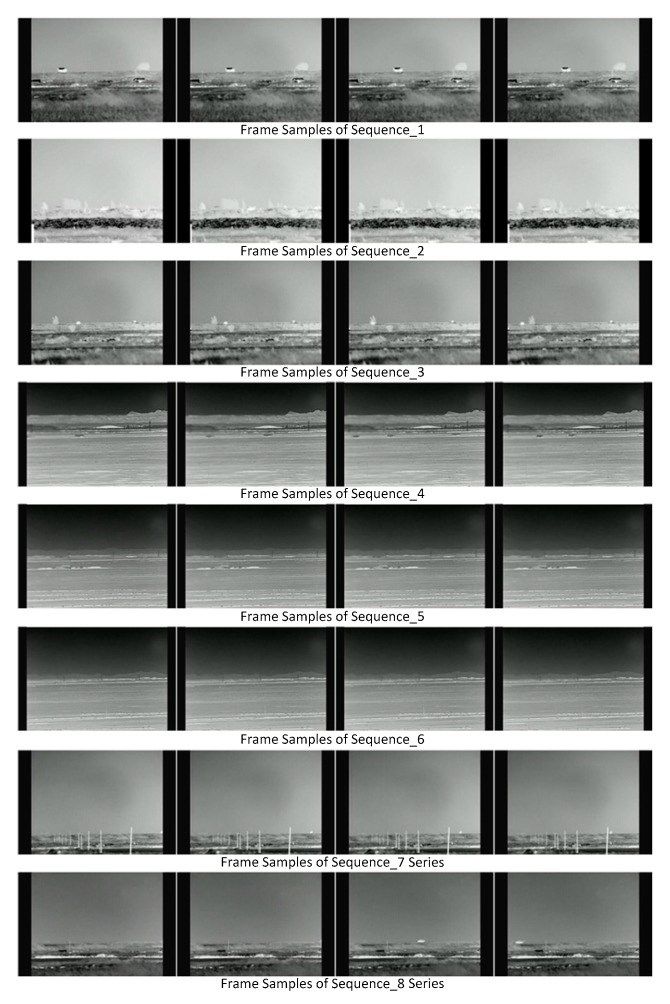
Frame samples in Remote Scene IR Dataset.

This dataset is composed of 1263 frames in 12 video sequences, and each frame was manually annotated with pixel-wise foreground. Frame samples of this dataset are shown in Figure 7. The frames in each video sequence are resized to 480 × 320, and they are provided in .BMP format. These IR video sequences represent several BS challenges, including dynamic background, ghosts, camera jitter, camouflage, noise, high and low speeds of foreground movement, etc. This dataset is described in Table 4 like the introduction of the previous datasets in Table 1. The challenges represented in each video sequence are listed in Table 5.

Sequence_1: In this sequence, foreground exists from the first frame. This is used to evaluate the capability of BS algorithms to handle ghosts. There is also waving grass, a typical dynamic background, in the frames of this sequence.

Sequence_2: Besides the challenges of ghost and dynamic background, there is a long duration camouflage. Foreground moves into a background region which has very similar color and texture with foreground.

Sequence_3: Challenges of ghost, dynamic background and camouflage are represented in this sequence. Different from Sequence_2, there is a short duration camouflage in this sequence which lasts from frame 77 to 102.

Sequence_4: This is a multi-foreground scene. Because of device noise, the left part of each frame in this sequence is blurred. There are also camera jitters in frames 39, 74, 85, 92, 98, etc.

Sequence_5: This sequence is used to detect small and dim foregrounds. Like sequence 4, there is also device noise in this sequence.

Sequence_6: Besides the challenges of device noise, small and dim foreground, there are also camera jitters in frames 18, 21, 24, 30, 108, etc.

Sequence_7 series: Sequences_7-1, Sequences_7-2 and Sequences_7-3 are the same videos with different frame sample rate, which are used to evaluate the capability of BS to handle low speed foreground movement. In Sequence_7-1, the speed is 1 pixel/frame. In Sequences_7-2 and Sequences_7-3, the speeds are respectively below and above 1 pixel/frame: 0.6 and 1.38 pixel/frame.

Sequence_8 series: Sequence_8-1, Sequences_8-2 and Sequences_8-3 are also the same videos with different frame sample rates. Contrary to the Sequence_7 series, these sequences are used to evaluate the capability of BS to handle high speed foreground movement. In Sequence_8-1, the speed is 1 self-size/frame. In Sequences_8-2 and Sequences_8-3, the speeds are respectively below and above 1 self-size/frame: 0.75 and 1.25 self-size/frame.

## 5. Experimental Setup

In the evaluation experiments, we attempted to select the most influential BS algorithms, the important BS algorithms from each category according to the taxonomy provided by [42], and the state-of-the-art BS algorithms.

The algorithms in the basic method category, such as frame difference, are very simple ways to detect moving objects. AdaptiveMedian [10] and Sigma-Delta [57] are relatively new approaches in this category. Bayes [16], an influential approach, is one of the earliest works which adaptively selects parameters (background learning rate) and adopts multiple features. Texture [45] is the first work to utilize discriminative texture features in the background model. SOBS [17] proposed a neural network method in which the background is modeled in a self-organizing manner. Gaussian [11], GMM1 [69], GMM2 [63] and GMM3 [15] are statistics-based approaches using a Gaussian model which is an important and influential model in lots of computer vision fields. Even though the Gaussian model is important, it is still not always perfectly corresponds to the real data because it is tightly coupled with the underlying assumptions. On the other hand, non-parametric models are more flexible, and are data dependent [17]. Codebook [65,66], GMG [13], KDE [61], KNN [14], ViBe [59] and PBAS [52] etc. are non-parametric BS approaches. ViBe and PBAS, two of the state-of-the-art approaches, proposed regional diffusion and eaten-up, respectively, which are effective mechanisms to increase the robustness of BS by sharing information between the neighborhood pixels as mentioned in Section 3.2. PBAS also proposed adopting a feedback loop to adaptively adjust the parameter for each pixel separately at runtime. PACWS, which is also one of the state-of-the-art BS algorithms is a hybrid of Codebook [65,66] and ViBe [59], and it also adopts a feedback loop to adjust parameters. The implementations and parameter settings of these evaluated BS algorithms are presented in Section 5.1 and Section 5.2. This evaluation paper is described in Table 6 like with the introduction of the previous evaluation papers in Table 2. All the evaluated BS algorithms were implemented based on Opencv-2.4.9.

### 5.1. Implementation of BS Algorithms

In this evaluation, we tried to keep the implementations of BS consistent with the description in BS papers, and performed few modifications. For a fair comparison, we first removed any post-processing described in BS papers, and then evaluated these BS algorithms without and with post-processing, respectively. Also for a fair comparison of memory and processor requirements, we removed the parallel threads described in BS papers. The six issues of these 16 BS algorithms are detailed in Table 7, and the modifications based on the original BS papers are presented as follows:

Bayes: We removed the morphological operation in the Section 3.3 of [16].

Codebook: We used the implementation in legacy module of Opencv2.4.9, which is a simplification of the Codebook BS algorithm [65,66]. This implementation applies minus to measure the similarity between pixel and its BG model and employs a bit-wise OR operation for multi-channel. In the experiments, YUV color feature are adopted for this algorithm.

GMG: We removed filter and connected components in Section D of [13].

GMM3: We removed the shadow detection in Section 2 of [15].

KNN: We removed the shadow detection in Section 2 of [14].

PBAS: We only applied one thread to run this algorithm on three channels instead of three parallel threads in Section 3.5 of [52].

SOBS: We removed the shadow detection in Section B of [17].

### 5.2. Parameter Settings of BS Algorithms

For the parameter settings of the evaluated BS algorithms, we also tried to keep them consistent with the values in BS papers. The parameter settings in the experiments are listed in Table 8.

### 5.3. Statistical Evaluation Metrics

**Figure 8:**
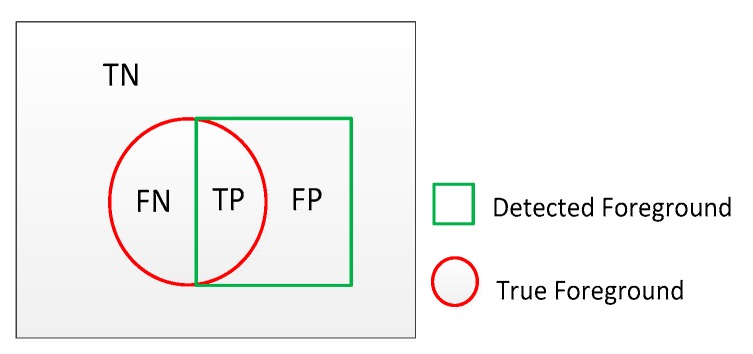
True and detected foreground in background subtraction.

Background subtraction is considered as a binary classification problem: a pixel is labeled as background or foreground. As shown in Figure 8, the circle and square respectively represent the true and detected foreground. TP is the number of true positives, TN is the number of true negatives, FN is the number of false negatives and FP is the number of false positives. Three important evaluation metrics are computed with Equations (3)–(5):

(3) Precision= TP/(TP+FP)

(4) Recall= TP/(TP+FN)

(5) F−measure = 2* Recall* Precision/(Recall+ Precision)

Precision can be seen as a metric of exactness or quality, whereas recall is a metric of completeness or quantity. For a better BS algorithm, the scores of precision and recall should be both high, but there is an inverse relationship between precision and recall, where it is possible to increase one at the cost of reducing the other. The F-Measure which is a harmonic mean of precision and recall can be viewed as a compromise between precision and recall. It balances the precision and recall with equal weighs, and it is high only when both recall and precision are high. A higher score of F-Measure means that the performance of BS algorithm is better.

**Figure 9:**
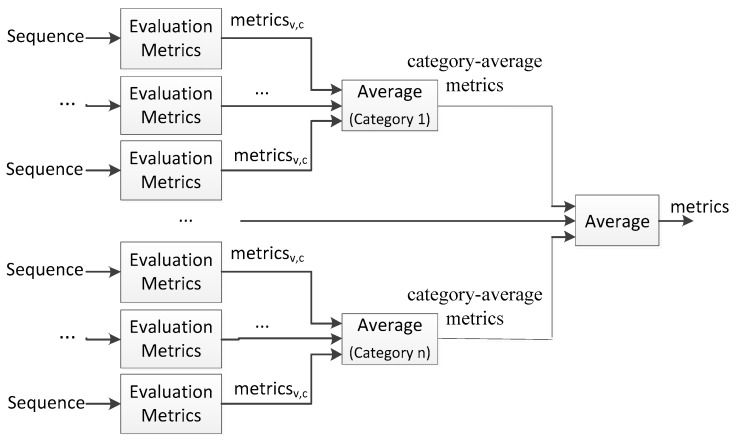
Evaluation metrics in CDW challenges.

In the CVPR CDW challenges, the evaluation metrics are average-based. The metrics for each sequence are firstly calculated. The category-average metrics for each category are computed from these metrics for all videos in a single category. The final metrics are also computed by averaging the category-average metrics. This calculation process of the evaluation metrics is presented in Figure 9. It is clear that there is a shortcoming of these average-based metrics. They are not suitable for situations where the number of frames in each video is unbalanced or the number of videos in each category is unbalanced.

**Figure 10:**
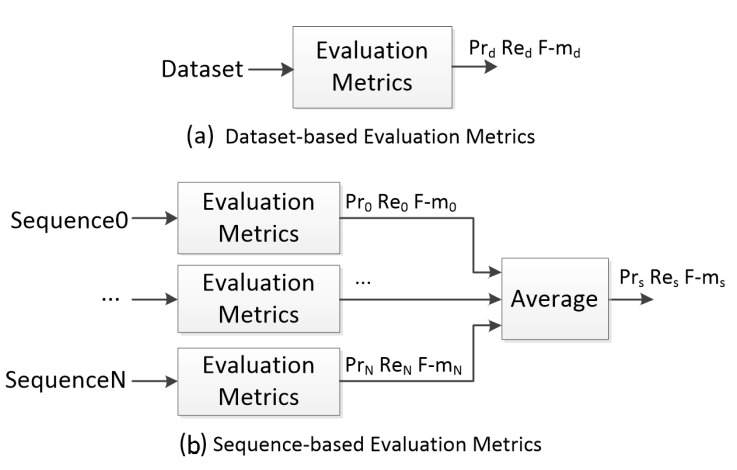
Dataset-based and sequence-based evaluation metrics.

Even though there is no category level in the Remote Scene IR dataset, the situation that the number of frames is unbalanced indeed exists. For example, the frame number of Sequence_6 is five times that of Sequence8_1. To overcome this problem, we also employ the overall-based metrics. We term these two kinds of metrics, which are shown in Figure 10, as sequence-based evaluation metrics (Pr_s_, Re_s_, F-m_s_) and dataset-based evaluation metrics (Pr_d_, Re_d_, F-m_d_), respectively. For sequence-based evaluation metrics which are similar to the metrics in the CDW challenge, evaluation metrics are firstly calculated for each sequence independently, and then the average is calculated as the final evaluation metrics. Dataset-based evaluation metrics are computed across all the frames in the whole dataset.

### 5.4. Rank-Order Rules

Two kinds of rank-orders (named as R and RC) are given in the CDW challenge. Like the average-based metrics, the rank-order R is also not suitable for the situation where the number of videos in each category is unbalanced. R and RC are both calculated in the same process: BS algorithms are firstly ranked based on each evaluation metric independently, and the average of these ranks is calculated as the final rank. Actually it is difficult to be certain that the process in which the rank of each metric is firstly calculated is better than the process in which the average of metrics is firstly calculated.

**Figure 11:**
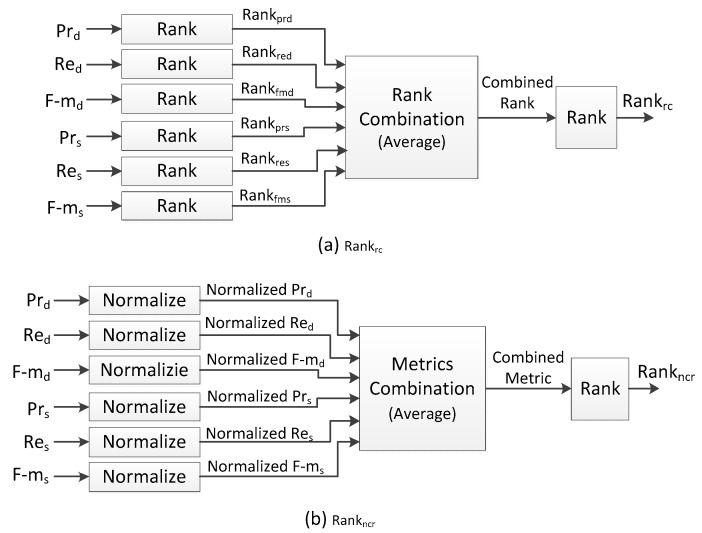
Two proposed rank-order rules of BS algorithms.

In the following evaluation experiments, we attempt to employ both of the two calculation processes in which rank and average is respectively firstly calculated. These two rank-orders named Rank_rc_ and Rank_ncr_ are not only based on the sequence-based evaluation metrics (Pr_s_, Re_s_, F-m_s_), but also the dataset-based evaluation metrics (Pr_d_, Re_d_, F-m_d_). Figure 11a is the overview of Rank_rc_. Firstly BS algorithms are ranked based on each evaluation metric independently, and the average of these ranks is calculated as the combined rank. The BS algorithms are finally ranked based on this combined rank. Figure 11b is the overview of Rank_ncr_. Each evaluation metric is normalized in the range [0, 1], and the average of these normalized metrics is firstly calculated as a combined metric. The BS algorithms are ranked based on this combined metric.

### 5.5. Other Evaluation Metrics

To compare BS algorithms in the intrusion detection context, [70] proposed a multi-level evaluation methodology including pixel level, image level and sequence level. Besides the aforementioned evaluation metrics precision (Pr), recall (Re) and F-Measure (F-m), [70] also adopted the average error number (Err) and standard deviation (SD). To locate the detection errors, [70] proposed D-Score. The pixel of the pixel S(x,y) is computed as the Equation (6).

(6) $$D-Score(S(x,y))=\exp(-\ln{\left(2DT(S(x,y))-5/2\right)}^{2})$$

where DT(S(x,y) is given by the minimal distance between the pixel S(x,y) and the nearest reference point (by distance transformation algorithm). A good D-Score has to tend to 0. The D-Score on a given frame is the mean of D-Score on each pixel, and the D-Score on a given sequence is also the mean of D-Score on each frame.

In [70], Pr, Re and F-m were used in all levels of its proposed multi-level evaluation methodology, and Err, SD and D-Score were used only in the pixel-based level. Different from the intrusion detection context, the true foreground exists almost in each frame of the Remote Scene IR Dataset, which means that both FP and TN are always 0 in the frame level and sequence level, even FN is also 0 in these two levels. According to Equations (3)–(5), Pr, Re and F-m in the frame and sequence levels are always 1 which cannot represent the real performance of BS, so we only employ pixel level metrics (Pr, Re, F-m, Err, SD and D-Score) in [70] for our evaluation experiments. Actually, the two kinds of metrics introduced in Section 5.3 are both pixel-level metrics, and they will be used in all the experiments. For Err, SD and D-Score, we will try to adopt them for the overall evaluation of BS algorithms in Section 6.1.

## 6. Experimental Results

In this section, the overall experimental results and the effects by post-processing are presented. Proper evaluation metrics or criteria are selected to evaluate the capability of the evaluated BS algorithms to handle various challenges. The computational load and memory usage required by each BS algorithm are also presented in this section.

### 6.1. Overall Results

The evaluation metrics and rank-orders of BS algorithms are listed in Table 9. Because of the characteristics of the remote scene IR video sequence, this evaluation result is different with that of the previous evaluation works.

It is noted that two recent BS algorithms SOBS and ViBe which employ regional diffusion and a traditional BS algorithm Sigma-Delta perform best. All three of these BS algorithms adopt color features. The BS algorithms PCAWS and Texture which adopt texture features perform worst because of the insufficient texture information in the remote scene IR video sequence. The evaluation metrics Err, SD and D-Score of the BS algorithms are also calculated according to [70] and are shown in Table 10.

It is noted that the results presented in this table are different from those presented in Table 9 and neither are consistent with what we directly observe from the detected foreground masks. For example, PCAWS and KDE give good results in Table 10 but bad results in Table 9. We argue that there are two reasons which could explain the ‘good’ results in Table 10. First, Err, SD and D-Score are one-sided metrics which only consider the errors of the detection including FN and FP, not the whole detection including FN, FP and TN, TP. They cannot present the real performance of the BS algorithm in some situations. We take the Err of PCAWS as an example. This small value of Err (FN and FP) is due to the small moving object (including FN) in the remote scene and the worse performance of PCAWS which detects little foreground (including FP), not due to the ‘good’ performance of PCAWS. This situation also can be illustrated by Figure 8 in which the circle and square are both very small. Second, for D-Score, each error cost depends to the distance with the nearest corresponding pixel in the ground-truth, and the penalty applied to the medium range is heavier than that applied to the short or long range [70]. According this evaluation criterion based on the range, [70] implemented D-Score with a tolerance of 3 pixels from the ground-truth. Also due to the small moving objects in remote scene, actually the errors with 3 pixels range would really affect the detection process, so the Err, SD and D-Score cannot effectively present the real performance of BS algorithms in this proposed dataset, therefore in the following experiments, Err, SD and D-Score would not be adopted for evaluation. In order to assess the difficulty that each IR video sequence poses to the evaluated BS algorithms, we calculate the average of all the evaluated BS algorithms’ F-m_s_ for each sequence, and rank the difficulty according to this average value. The results are listed in Table 11 which shows that it is much more difficult to subtract background on the video sequences presenting challenges of small and dim foreground, camouflage and low speed of foreground movement.

### 6.2. Post-Processing

After BS post-processing approaches that detect foreground masks including median filter, morphological operation and shadow removal are commonly used to improve the performance of BS. Because of the inexistence of shadow in the Remote Scene IR Dataset, we only focused on the median filter and morphological operation. In this post-processing experiment, firstly a median filter with a 3 × 3 window was employed on the detected foreground masks. Then a morphological operation was employed on the detected foreground masks, including opening operation and closing operation within one iteration with a 3 × 3 window.

Table 12 illustrates the results of the BS with median filters (BS + M), and Table 13 illustrates the results of the BS with median filters and morphological operation (BS + MM). Most of BS algorithms benefit from these post-processing approaches, and the improvements of performance are presented in Table 14 and Table 15.

Due to the benefit from median filter, F-m_d_ and F-m_s_ are improved by an average of 0.0369 and 0.0329, respectively. PBAS and Codebook get the most benefit. F-m_d_ of PBAS is increased by 0.1073. Rank_rc_ of PBAS is improved by 1. F-m_s_ of Codebook is increased by 0.1323. Rank_rc_ and Rank_ncr_ of Codebook are respectively improved by 3 and 2.

Due to the benefit from median filter and morphological operation, F-m_d_ and F-m_s_ are improved by an average of 0.0523 and 0.0479, respectively. PBAS and Codebook also get most benefit. F-m_d_ of PBAS is increased by 0.1922. Rank_rc_ and Rank_ncr_ of PBAS are respectively improved by 1 and 2. F-m_s_ of Codebook is increased by 0.2265. Rank_rc_ and Rank_ncr_ of Codebook are respectively improved by 4 and 5.

### 6.3. Camera Jitter

**Figure 12:**
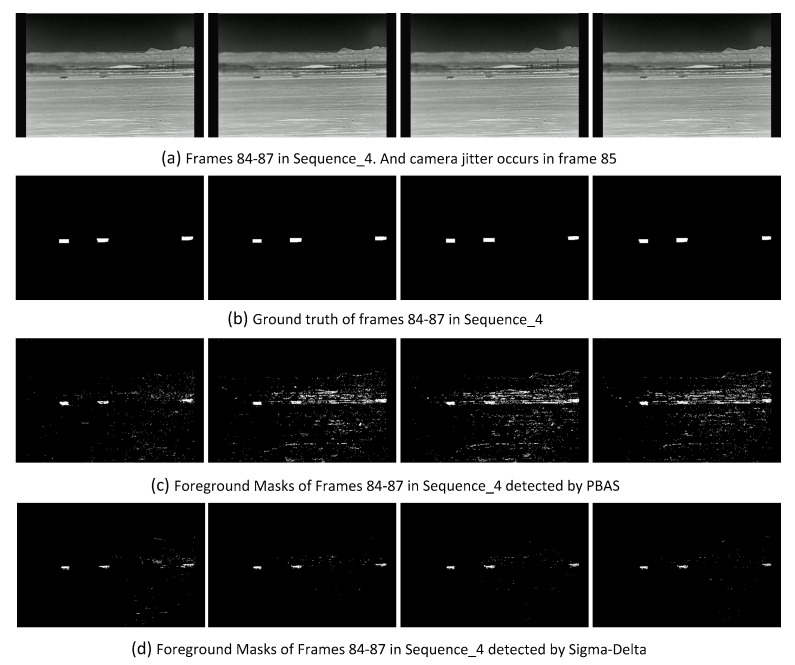
Comparsion of the results detected by different BS algorithms for the challenge of camera jitter.

In many situations, camera jitter is often encountered, which poses a great challenge for BS. When it occurs, FP is increased significantly in the next several frames. Take the camera jitter in frame 85 of Sequence_4 as an example, Figure 12 shows frames 84 to 87, their ground truth and the foreground masks detected by PBAS and Sigma-Delta, and it is obvious that camera jitter could introduce much more FP in the some BS algorithms. It is easy to understand that a BS algorithm with a strong capability to handle this challenge, should introduce few FP, but as a special case, the few FP after camera jitter is caused by the weak capability of detection. As an extreme example, there are few foreground pixels (including TP and FP) detected by PCAWS in each frame of Sequence_4. It is clear that the few FP after camera jitter is not caused by the strong capability of PCAWS to handle this challenge, so we evaluate the capability of BS to handle camera jitter not only based on the increase of FP, but also based on the detected foreground pixels (sum of FP and TP). Suppose the FP_i_ and TP_i_ are respectively FP and TP of the frame i and the cameral jitter occurs in frame t, the evaluation metric P_cj_ employs first n frames after camera jitter, which is defined by Equation (7). A small value of P_cj_ means a strong capability to handle camera jitter. We try to only focus on the impact caused by camera jitter, and take a small value 3 for n:

(7) $$P_{cj}=\sum_{i=t}^{t+n-1}\frac{FP_{i}-FP_{t-1}}{FP_{i}+TP_{i}}\;\mathrm{where}\;n=3$$

In this experiment, 10 distinct camera jitters (frames 39, 74, 85, 92, 98 of Sequence_4 and frames 18, 21, 24, 30, 108 of Sequence_6) were employed to evaluate the capability of BS to handle this challenge. Table 16 presents the average P_cj_ of these 10 camera jitters for each evaluated BS algorithm. Adaptive Median, Bayes as well as ViBe perform best, and Codebook, PBAS as well as SOBS perform worst. This evaluation result is consistent with what we directly observe from the detected foreground masks.

### 6.4. Ghosts

**Figure 13:**
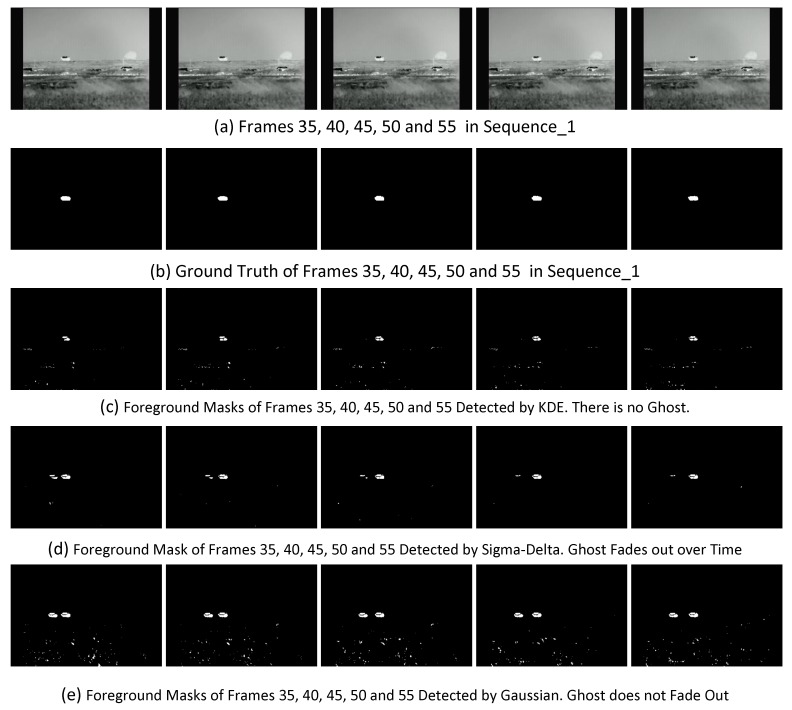
Comparison of the ghosts detected by different BS algorithms.

When a foreground exists from the first frame or a static foreground starts moving, there would be an artifact ghost left because the pixels of the foreground are involved in the BG model initiation. A ghost is a set of connected points, detected as in motion but not corresponding to any true foreground [71]. In the Remote Scene IR Dataset, Sequence_1, Sequence_3 and Sequence_4 represent the ghost challenge. The capability of each algorithm to handle this challenge can be evaluated by directly observing the detected foreground masks. BS algorithms including Bayes, GMG, KDE, KNN which adopt density feature or probability measurement and BS algorithm PBAS perform best. There is no ghost in the foreground masks detected by these algorithms. For SOBS, ghosts do not appear in the foreground masks of Sequence_3 and Sequence_4, but appear in the foreground masks of Sequence_2 in which the foreground has a big size. In the foreground masks of GMM3, Sigma-Delta and ViBe, ghosts appear but they obviously fade out over time. The order of fade rate is Sigma-Delta, GMM3, ViBe. Texture and PCAWS are not evaluated for the ghost challenge because of the poor results on these three sequences. There are ghosts in each foreground mask detected by the remaining BS algorithms, which perform worse at handling this challenge. Figure 13 shows the three kinds of Ghost results detected by KDE, Sigma-Delta and Gaussian, respectively.

### 6.5. Low Speed of Foreground Movement

Low speed of foreground movement is a challenge of BS, and it is very common in remote scenes. As described in Section 1.1, when the foreground moves with a low speed, it is difficult to distinguish foreground pixels. In the Remote Scene IR Dataset, Sequence_7 series represents this challenge. The speeds in Sequence_7-1, Sequence_7-2 and Sequence_7-3 are respectively 1 pixel/frame, 0.6 pixel/frame and 1.38 pixel/frame.

To only focus this challenge which poses difficulty to distinguish foreground pixels, we selected evaluation metric recall to evaluate the capability of BS to handle this challenge. Table 17 shows Re_s_ of each BS algorithm tested on the Sequence_7 series. The averages of all the evaluated BS algorithms’ Re_s_ are 0.2226, 0.2397 and 0.2438, respectively, for Sequence_7-2, Sequence_7-1 and Sequence_7-3. This means that for this challenge, the slower the foreground moves, the fewer foreground pixels are detected. It is noted that Re_s_ of Bayes and KNN on Sequence_7-2 are much smaller than them on Sequence_7-1 and Sequence_7-3. This means that when the speed is below 1 pixel/frame, the performance of Bayes and KNN will decrease significantly. Table 17 also shows that GMM3 as well as PBAS perform best for this challenge, and GMM2, KDE as well as PCAWS which hardly detect foreground pixels perform worst for this challenge.

### 6.6. High Speed of Foreground Movement

**Figure 14:**
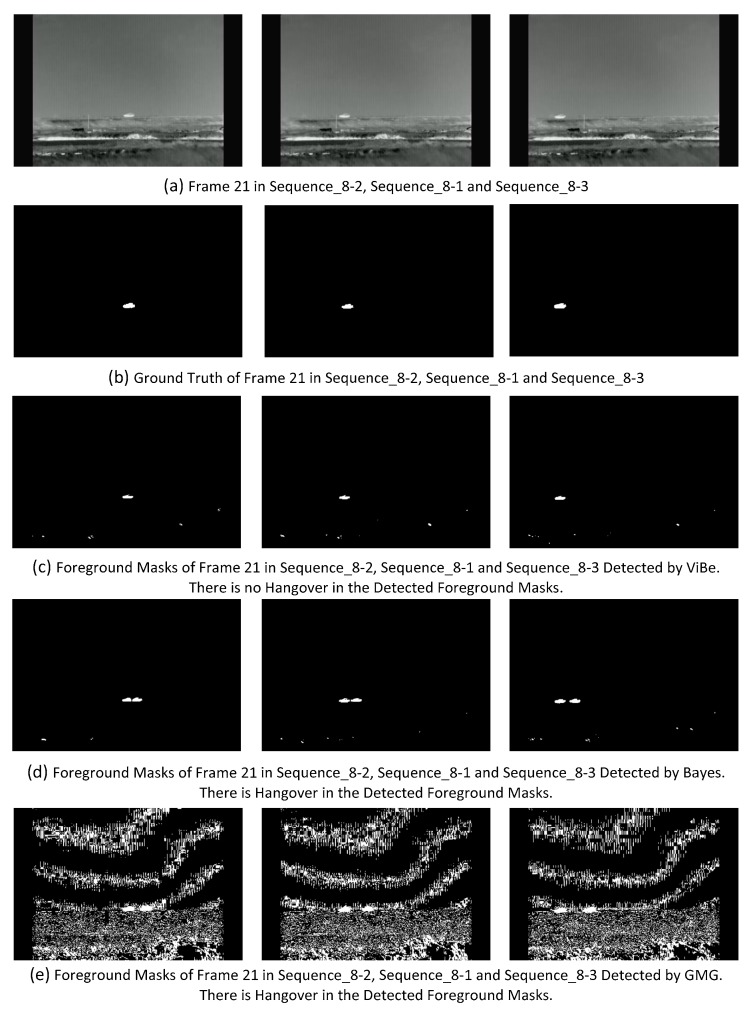
Comparison of the results detected by different BS algorithms for the challenge of high speed foreground movement.

High speed of foreground movement is also a challenge of BS which is not mentioned in the previous BS works. As described in Section 1.1, if the foreground moves with high speed, there would be a hangover. In Remote Scene IR Dataset, Sequence_8 series represents this challenge. The speeds in Sequence_8-1, Sequence_8-2 and Sequence_8-3 are 1 self-size/frame, 0.75 self-size/frame and 1.25 self-size/frame, respectively. When the speed of foreground movement is enough high, some BS algorithms produce hangover which is FP. By observing the foreground masks of Sequence_8 series detected by each evaluated BS algorithm, we found that only Bayes and GMG produce hangover. The faster the foreground moves, the longer the distance between the detected foreground and the hangover is. Figure 14 shows the different results detected by ViBe, Bayes and GMG for the challenge of high speed foreground movement. Figure 14c shows the foreground masks of frame 21 in Sequence_8-2, Sequence_8-1 and Sequence_8-3 detected by ViBe without hangover. Figure 14d,e show foreground masks of the same frames detected by Bayes and GMG, and there are hangovers appeared. In the foreground masks detected by GMG, besides handover, there is also other FP, which is not caused by the challenge of high speed of foreground movement. We only focus on the hangover caused by this challenge. It is noted that hangover fades out over time in the foreground masks detected by GMG, but cannot fade out in the foreground masks detected by Bayes.

### 6.7. Camouflage

Camouflage is a challenge of BS which is caused by foreground that has similar color and texture as the background. There is a long duration of camouflage in Sequence_2, and the foreground moves into a background region which has very similar color and texture as the foreground. Table 18 presents the evaluation metric F-m_s_ of each evaluated BS algorithm. Two recent algorithms PBAS, SOBS, and a traditional algorithm, Codebook, perform the best and they benefit greatly from the post-processing. GMM2, KDE and PCAWS perform the worst. They hardly detect foreground pixels, and do not gain any benefit from post-processing.

### 6.8. Small Dim Foreground

Small and dim foregrounds are also challenges in BS which are common in remote scenes. There are small and dim foregrounds in Sequence_5 and Sequence_6. Table 19 presents the average F-m_s_ of all the evaluated BS algorithms for these two sequences. It is noticed that median filter improves the performance of BS but morphological operation decreases the performance of BS, so we only focus on the results of BS and BS with median filter for this challenge. Table 20 depicts the average F-m_s_ of these two sequences for each BS algorithm. Sigma-Delta, KNN, Gaussian as well as Bayes perform best, and when the median filter is employed, Codebook, GMM1, PBAS as well as Bayes get the most benefit and perform best.

### 6.9. Computational Load and Memory Usage

Because computational load and memory usage are crucial for the real-time video analysis and tracking applications and embedded systems, it is necessary to evaluate them for BS algorithms. In this paper, all the evaluation experiments were conducted on a personal computer with an Intel Core i7-3740QM 2.7 GHz × 8 CUP, 16 GB DDR3 RAM and Ubuntu 14.04 LTS.

Resident Set Size (RSS) and Unique Set Size (USS) were adopted to evaluate the memory usage of the BS algorithms. CPU occupancy and execution time were adopted to evaluate the computational load of BS algorithms. Table 21 presents the maximum USS, maximum RSS, average CPU occupancy and average execution time. Adaptive Median, Gaussian and Sigma-Delta consume the least memory. Codebook, KDE and ViBe have the minimum computational complexity.

### 6.10. Extensional Evaluation with BGSLibrary

BGSLibrary [1] is a very powerful library with many BS algorithms already implemented. We conducted an extensional evaluation on the BS algorithms from this library. We selected the BS algorithms which are not evaluated in the previous experiments of this paper, which are listed in Table 22. The BS algorithms in this library are implemented by many contributors. After checking the implementations of the selected algorithms in this library, it is found that the results of some algorithms cannot be evaluated using the same metrics and the implementations of some algorithms are different from the descriptions in the original papers. For example, the foreground mask detected by the MultiCue algorithm [72] is not the binary mask; the update of the BG model in the Texture2 algorithm [45] is different from the update in the original paper. Therefore we just conducted an overall evaluation experiment on these BS algorithms. The comprehensive evaluation of the BS algorithms from BGSLibrary [1] on the proposed Remote Scene IR dataset will be conducted after a clear grasp of their detailed implementation.

We ported 24 BS algorithms from BGSLibrary, and made some modifications to ensure that these BS algorithms can be evaluated in the same context as the previous experiments in this paper. For example, we removed the median filter from AdaptiveSelectiveBGLearning, adopted the foreground mask with single channel instead of foreground image with three channels in FuzzyGaussian [11,73], TextureMRF [46], GMM-Laurence [40], SimpleGaussian [28] and FuzzyAdaptiveSOM [64], and also some other modifications. The dataset-based evaluation metrics of these BS algorithms are presented in Table 22, including the results of BS, BS with median filters (BS + M) and BS with median filters and morphological operation (BS + MM). It is found that the conclusion of this extensional evaluation is similar with that of the previous experiments in Section 6.1. The performance of these BS algorithms on this dataset is different from the performance on other datasets. There are also some state-of-the-art BS algorithms such as SuBSENSE [68] and LOBSTER [48] which do not perform so well, because they only employ texture features; and there are also some simple basic algorithms such as AdaptiveBGLearning which perform well.

## 7. Discussion

In this paper, we proposed a challenging Remote Scene IR dataset which represents several challenges of BS. We improved the rank-order rules in CVPR CDW challenge to overcome the unbalance and uncertainty problems. We also proposed a selection of proper evaluation criteria to evaluate the capabilities of BS to handle various BS challenges, instead of using the same evaluation criteria for all the evaluations of capabilities.

In the evaluation experiments, it is found that due to the characteristics of the proposed dataset, the performance of BS algorithm on this dataset is different with the performance on other datasets. The PCAWS and Texture which only employ texture features perform worse, even though PCAWS is one of the state-of-the-art BS algorithms and performs well on other datasets. One simple basic BS algorithm, Sigma-Delta, performed unexpectedly well.

In extension evaluation experiments on the BS algorithms from the BGSLibrary, the same conclusions were drawn. The BS algorithms, including the state-of-the-art methods which only employ texture features, perform worse, while some simple basic BS algorithms perform well. However the extended evaluation experiments were not as comprehensive as the evaluation experiments, therefore, a double check on the implementations of the BS algorithms in the BGSLibrary and a comprehensive evaluation experiment on this proposed dataset are future works.

Remote scene IR video sequences poses enormous difficulties to background subtraction, and F-m_d_ of the best BS algorithm with post-processing in the evaluation experiments and the extension evaluation experiment were only 0.5398 and 0.511, respectively, which cannot meet the requirement of some video analysis and tracking systems or applications. According to the results of the evaluation experiments, the algorithms SOBS and ViBe which employ regional diffusion and the algorithm Sigma-Delta perform well, and ViBe, Sigma-Delta also require a small computational load and low memory consumption, but Sigma-Delta performs worse when handling the challenge of camera jitter. Both Sigma-Delta and ViBe perform worse at handling the challenges of camouflage and low speed of foreground movement. It is also found that even though the overall result of PBAS was not as good as the results of Sigma-Delta and ViBe, PBAS has good capability to handle the challenge of camera jitter due to its eaten-up mechanism and good capability to handle the challenges of camouflage and low speed of foreground movement due to its feedback loop mechanism. These good capabilities can be explained by the roles of these new mechanisms which have been introduced in Section 3.2. We also argue that a reason why the overall result of PBAS is not so good is that PBAS adopts the gradient magnitude as the feature which is weak information in IR remote scenes.

Regarding the final purpose of developing an effective and efficient BS algorithm for IR remote scenes, it is clear that ViBe could be improved by adding a feedback loop to adaptively adjust parameters, or Sigma-Delta could be improved by adding a region diffusion or eaten-up mechanism and also adding a feedback loop. We can also try to remove the gradient magnitude feature from PBAS and only retain the color feature. Compared to ViBe and Sigma-Delta, PBAS would still have a heavy computer load and memory usage, even if the gradient magnitude feature were removed.

## Figures and Tables

**Table 1:** Introduction of the datasets recently developed for background subtraction.

Datasets	Type	Ground Truth	Challenges
SABS	Synthetic	Pixel-wise FG and Shadow	Dynamic Background, Bootstrapping, Darkening, Light Switch, Noisy Night, Shadow, Camouflage, Video Compression
CDW2012	Realistic	Pixel-wise FG, ROI and Shadow	Dynamic BG, Camera Jitter, Intermittent Motion, Shadow, Thermal
CDW2014	Realistic	Pixel-wise FG, ROI and Shadow	Dynamic BG, Camera Jitter, Intermittent Motion, Shadow, Thermal, Bad Weather, Low Frame Rate, Night, PTZ, Air Turbulence
BMC	Synthetic and Realistic	Pixel-wise FG for Part of Video Sequences	Dynamic Background, Bad Weather, Fast Light Changes, Big foreground
MarDCT	Realistic	Pixel-wise FG for Part of Video Sequences	Dynamic Background, PTZ
CBS RGB-D	Realistic	Pixel-wise FG	Shadow, Depth Camouflage
FDR RGB-D	Realistic	Pixel-wise FG for Part of Video Sequence	Low Lighting, Color Saturation, Crossing, Shadow, Occlusion, Sudden Illumination Change

**Table 2:** Introduction of recent evaluation and review papers on background subtraction.

Papers	Dataset	Evaluation Metrics
Brutzer et al.	SABS	F-Measure, PRC
Goyette et al.	CDW2012	Recall, Specificity, FPR, FNR, PWC, F-Measure, Precision, RC, R
Wang et al.	CDW2014	Recall, Specificity, FPR, FNR, PWC, F-Measure, Precision, RC, R
Vacavant et al.	BMC	F-Measure, D-Score, PSNR, SSIM, Precision, Recall
Sobral et al.	BMC	Recall, Precision, F-Measure, PSNR, D-Score, SSIM, FSD, Memory Usage, Computational Load
Dhome et al.	Sequences from LIVIC SIVIC Simulator	△-Measure, F-Measure
Benezeth et al.	A collection from PETS, IBM and VSSN	Recall, PRC, Memory Usage, Computational Load
Bouwmans	No	No

**Table 3:** Specifications of the MWIR sensor.

Detector Material: HgCdTe	NETD: <28 mk
Array Size: 640 × 512	Pixel Size: 15 μm
Diameter: 200 mm	Focus length: 400 mm
Wavelength Range: 3~5 μm	F/#: 4
Focusing Time: <1 s	Average Transmittance: >80%
FOV: 15.2° (Wide), 0.8° (Narrow)	Distortion: <7% (Wide), <5% (Narrow)
Data Bus: CameraLink or Fiber	Control Bus: CameraLink or RS422
Storage Temperature: −45~+60 °C	Operating Temperature: −40~+55 °C
Input Power: DC24 V ± 1 V, ≤35 W@20 °C	

**Table 4:** Introduction of the Remote Scene IR dataset.

Dataset	Type	Ground Truth	Challenges
Remote Scene IR Dataset	Realistic	Pixel-wise FG	Dynamic BG, Camera Jitter, Camouflage, Device Noise, High and Low speeds of Foreground Movement, Small and Dim Foreground, Ghost

**Table 5:** Challenges represented in each video sequence of the Remote Scene IR Dataset.

Sequences	Challenges
Sequences_1	Ghost, Dynamic Background
Sequences_2	Dynamic Background, Long Time Camouflage
Sequences_3	Ghost, Dynamic Background, Short Time Camouflage
Sequences_4	Ghost, Device Noise, Camera Jitter
Sequences_5	Small and Dim Foreground, Device Noise
Sequences_6	Small and Dim Foreground, Device Noise, Camera Jitter
Sequence_7 series	Low Speed of Foreground Movement
Sequence_8 series	High Speed of Foreground Movement

**Table 6:** Introduction of this evaluation paper.

Evaluation papers	Datasets	Evaluation Metrics
This Paper	Remote Scene IR Dataset	Recall_d_, Precision_d_, F-Measure_d_, Recall_s_, Precision_s_, F-Measure_s_, Rank_rc_, Rank_ncr_, USS, RSS, Execution Time, CPU Occupancy

**Table 7:** Implementations of the evaluated BS algorithms.

BS	Initiation	Channels	Features	BG Model	Detection	Update
AdaptiveMedian	Several Frames (Detection in Initiation)	Bit-wise OR	RGB Color	Running Median	L1 Distance	Iterative
Bayes	One Frame	Bit-wise OR	Multi-feature Fusion (RGB Color & Color Co-occurrence)	Histogram	Probability	Hybrid (Selective & Iterative)
Codebook	Several Frames (No Detection in Initiation)	Bit-wise OR	YUV Color	Codeword	Minus	Selective
Gaussian	One Frame	Fusion	RGB Color	Statistics	L2 Distance	Iterative
GMG	Several Frames (No Detection in Initiation)	Fusion	RGB color	Histogram	Probability	Hybrid (Selective & Iterative)
GMM1	One Frame	Fusion	RGB Color	Statistics with Weights	L2 Distance	Hybrid (Selective & Iterative)
GMM2	One Frame	Fusion	RGB Color	Statistics with Weights	L2 Distance	Hybrid (Selective & Iterative)
GMM3	One Frame	Fusion	RGB Color	Statistics with Weights	L2 Distance	Hybrid (Selective & Iterative)
KDE	Several Frames (No Detection in Initiation)	Fusion	SGR Color	Density	Probability	FIFO
KNN	One Frame	Fusion	RGB Color	Density	L2 Distance	Random
PBAS	Several Frames (Detection in Initiation)	Bit-wise OR	Multi-feature Fusion (RGB Color & Gradient)	Features Value	L1 distance	Random
PCAWS	One Frame	Fusion	Multi-feature Fusion (RGB Color & LBSP)	Dictionary	L1 Distance	Random
Sigma-Delta	One Frame	Bit-wise OR	RGB Color	Temporal Standard Deviation	L1 Distance	Iterative
SOBS	Several Frames (Detection in Initiation)	Fusion	HSV Color	Neuronal Map	L2 Distance	Iterative
Texture	One Frame	Fusion	LBP	Histograms with Weights	Histogram Intersection	Hybrid (Selective & Iterative)
ViBe	One Frame	Fusion	RGB Color	Features Value	L1 Distance	Random

**Table 8:** Parameter settings of the evaluated BS algorithms.

BS Algorithm	Parameter Setting
AdaptiveMedian	Threshold = 20, InitialFrames = 20
Bayes	L_color_ = 64, N1_color_ = 30, N2_color_ = 50, L_co-occurrences_ = 32, N1_co-occurrences_ = 50, N2_co-occurrences_ = 80, α_1_ = 0.1, α_2_ = 0.005
Codebook	min = 3, max = 10, bound = 10, LearningFrames = 20
Gaussian	InitialFrames = 20, threshold = 3.5, α = 0.001
GMG	F_max_ = 64, α = 0.025, q = 16, p_F_ = 0.8, threshold = 0.8, T = 20
GMM1	Thredshold = 2.5, K = 4, T = 0.6, α = 0.002
GMM2	Thredshold = 2.5, K = 4, T = 0.6, α = 0.002
GMM3	Threshold = 3, K = 4, c_f_ = 0.1, α = 0.001, c_T_ = 0.01
KDE	th = 10e-8, W = 100, N = 50, InitialFrames = 20
KNN	T = 1000, K = 100, C_th_ = 20
PBAS	N = 35, #min = 2, R_inc/dec_ = 18, R_lower_ = 18, R_scale_ = 5, T_dec_ = 0.05, T_lower_ = 2, T_upper_ = 200
PCAWS	R_color_ = 20, R_desc_ = 2, t_0_ = 1000, N = 50, α = 0.01, λ_T_ = 0.5, λ_R_ = 0.01
Sigma-Delta	N = 4
SOBS	n = 3, K = 15, ε_1_ = 0.1, ε_2_ = 0.006, c_1_ = 1, c_2_ = 0.05
Texture	P = 6, R = 2, R_region_ = 5, K = 3, T_B_ = 0.8, T_P_ = 0.65, α_b_ = 0.01, α_w_ = 0.01
ViBe	N = 20, R = 20, #min = 2, Φ = 16

**Table 9:** Evaluation metrics and rank-orders of the evaluated BS algorithms.

BS	Pr_d_	Re_d_	F-m_d_	Pr_s_	Re_s_	F-m_s_	Rank_rc_	Rank_ncr_
AdaptiveMedian	0.3362	0.2600	0.2933	0.3445	0.5870	0.3971	7	4
Bayes	0.2138	0.2915	0.2467	0.3527	0.3908	0.3119	9	8
Codebook	0.5759	0.0559	0.1019	0.5425	0.1038	0.1482	11	12
Gaussian	0.5196	0.1944	0.2829	0.5680	0.2725	0.3471	4	5
GMG	0.4927	0.0210	0.0402	0.5000	0.0172	0.0324	14	14
GMM1	0.6838	0.0612	0.1124	0.7069	0.0720	0.1275	10	10
GMM2	0.1066	0.5165	0.1767	0.1138	0.6690	0.1744	8	9
GMM3	0.8121	0.0207	0.0403	0.8330	0.0181	0.0353	12	11
KDE	0.1976	0.1120	0.1429	0.1653	0.3086	0.1776	13	13
KNN	0.2408	0.4083	0.3029	0.3700	0.4690	0.3399	5	6
PBAS	0.6924	0.1279	0.2159	0.7724	0.1020	0.1716	6	7
PCAWS	0.0168	0.9475	0.0330	0.0058	0.0833	0.0108	16	16
Sigma-delta	0.4544	0.5553	0.4998	0.5200	0.5646	0.5037	1	1
SOBS	0.4548	0.3561	0.3995	0.4724	0.4673	0.4462	2	2
Texture	0.2431	0.0483	0.0806	0.3848	0.0584	0.0950	15	15
ViBe	0.3544	0.3526	0.3535	0.3791	0.63619	0.4318	3	3

**Table 10:** Evaluation metrics (Err, SD and D-Score) of the evaluated BS algorithms.

BS	Err %	SD %	D-Score 10^−2^	BS	Err %	SD %	D-Score 10^−2^
AdaptiveMedian	0.244	0.453	0.177	KDE	0.297	0.382	0.193
Bayes	0.177	0.139	0.116	KNN	0.156	0.148	0.098
Codebook	1.145	1.030	0.998	PBAS	0.882	0.403	0.781
Gaussian	0.417	0.686	0.339	PCAWS	0.136	0.132	0.01
GMG	3.793	0.917	3.461	Sigma-delta	0.141	0.171	0.091
GMM1	1.552	1.872	1.352	SOBS	0.184	0.196	0.125
GMM2	0.136	0.144	0.05	Texture	0.781	0.422	0.657
GMM3	5.487	2.80	4.892	ViBe	0.197	0.349	0.137

**Table 11:** Rank of difficulty that each IR video sequence poses to the evaluated BS algorithms.

	Ave. F-m_s_	Difficulty Rank		Ave. F-m_s_	Difficulty Rank
Sequence_1	0.3253	10	Sequence_7-1	0.2397	5
Sequence_2	0.1025	3	Sequence_7-2	0.2226	4
Sequence_3	0.3105	8	Sequence_7-3	0.2438	6
Sequence_4	0.2630	7	Sequence_8-1	0.3159	9
Sequence_5	0.0773	2	Sequence_8-2	0.3565	12
Sequence_6	0.0297	1	Sequence_8-3	0.3260	11

**Table 12:** Evaluation metrics and ranks of the evaluated BS with median filter.

BS + M	Pr_d_	Re_d_	F-m_d_	Pr_s_	Re_s_	F-m_s_	Rank_rc_	Rank_ncr_
AdaptiveMedian	0.3232	0.3323	0.3277	0.3273	0.6762	0.3919	6	5
Bayes	0.1644	0.5141	0.2491	0.3008	0.5549	0.3177	9	8
Codebook	0.5735	0.1051	0.1777	0.5322	0.2380	0.2805	8	10
Gaussian	0.5036	0.2631	0.3456	0.5407	0.4255	0.4193	4	4
GMG	0.4828	0.0655	0.1154	0.4909	0.0546	0.0897	14	14
GMM1	0.6826	0.0867	0.1539	0.6869	0.1496	0.2295	10	9
GMM2	0.0891	0.5932	0.1549	0.1032	0.5098	0.1617	11	12
GMM3	0.8344	0.0338	0.0650	0.8387	0.0336	0.0635	12	11
KDE	0.1847	0.1250	0.1491	0.1516	0.3866	0.1797	13	13
KNN	0.1871	0.6488	0.2905	0.3164	0.6249	0.3333	7	6
PBAS	0.6835	0.2117	0.3233	0.7579	0.1618	0.2500	5	7
PCAWS	0.0154	0.9484	0.0303	0.0053	0.0833	0.0100	16	16
Sigma-delta	0.4361	0.7082	0.5398	0.4918	0.6907	0.5261	**1**	**1**
SOBS	0.4441	0.5280	0.4824	0.4473	0.6169	0.4771	**2**	**2**
Texture	0.1493	0.0977	0.1181	0.3187	0.0956	0.1178	15	15
ViBe	0.3408	0.4553	0.3898	0.3626	0.6942	0.4294	**3**	**3**

**Table 13:** Evaluation metrics and ranks of the evaluated BS with median filter and morphological operation.

BS + MM	Pr_d_	Re_d_	F-m_d_	Pr_s_	Re_s_	F-m_s_	Rank_rc_	Rank_ncr_
AdaptiveMedian	0.3125	0.4434	0.3666	0.3098	0.6227	0.3803	6	6
Bayes	0.0909	0.5531	0.1561	0.2193	0.5777	0.2263	10	11
Codebook	0.5521	0.1520	0.2384	0.5069	0.3992	0.3747	7	7
Gaussian	0.4865	0.3552	0.4106	0.5127	0.5425	0.4565	**3**	**3**
GMG	0.4343	0.1253	0.1945	0.4395	0.1104	0.1492	11	12
GMM1	0.6559	0.1054	0.1817	0.6481	0.2251	0.2981	9	9
GMM2	0.0683	0.6016	0.1227	0.0872	0.4364	0.1411	14	13
GMM3	0.8260	0.0467	0.0884	0.8239	0.0608	0.1089	12	10
KDE	0.1675	0.1660	0.1667	0.1348	0.4266	0.1700	13	14
KNN	0.1130	0.7604	0.1968	0.2472	0.6180	0.2719	8	8
PBAS	0.6607	0.2952	0.4081	0.7320	0.2286	0.3310	5	5
PCAWS	0.0152	0.9556	0.0298	0.0052	0.0833	0.0098	16	15
Sigma-delta	0.4161	0.8228	0.5527	0.4674	0.7669	0.5676	**1**	**1**
SOBS	0.4245	0.6771	0.5218	0.4216	0.7371	0.4915	**2**	**2**
Texture	0.0896	0.1187	0.1021	0.2789	0.1114	0.1107	15	16
ViBe	0.3333	0.5788	0.4230	0.3500	0.6560	0.4298	4	4

**Table 14:** Improvement of BS performance caused by median filter.

BS + M	F-m_d_	F-m_S_
Average Improvement	0.0369	0.0329
Maximum Improvement	0.1073 (PBAS)	0.1323 (Codebook)

**Table 15:** Improvement of BS performance caused by median filter and morphological operation.

BS + MM	F-m_d_	F-m_S_
Average Improvement	0.0523	0.0479
Maximum Improvement	0.1922 (PBAS)	0.2265 (Codebook)

**Table 16:** Capability of the evaluated BS algorithms to handle camera jitter.

BS	AVE. P_cj_	BS	AVE. P_cj_
AdaptiveMedian	−0.8732	KDE	0.0030
Bayes	−0.4557	KNN	0.3918
Codebook	1.2910	PBAS	1.1581
Gaussian	0.9122	PCAWS	0.1000
GMG	0.3183	Sigma-Delta	0.3096
GMM1	0.8081	SOBS	1.1807
GMM2	0.2985	Texture	0.8852
GMM3	0.5778	ViBe	−0.8261

**Table 17:** Re_s_ of the evaluated BS algorithms tested on Sequence_7 series.

	Sequence_7-1	Sequence_7-2	Sequence_7-3
AdaptiveMedian	0.3137	0.3157	0.3173
Bayes	0.3048	0.1358	0.4321
Codebook	0.6683	0.7034	0.6512
Gaussian	0.5853	0.5679	0.6069
GMG	0.6926	0.5737	0.7391
GMM1	0.7315	0.7358	0.7389
GMM2	0.0006	0.0070	0.0002
GMM3	**0.8646**	**0.8590**	**0.8679**
KDE	0.0935	0.0984	0.0981
KNN	0.2424	0.1004	0.3442
PBAS	**0.8250**	**0.8369**	**0.8331**
PCAWS	0	0	0
Sigma-Delta	0.5211	0.4307	0.5720
SOBS	0.5581	0.5659	0.5542
Texture	0.2352	0.1576	0.3036
ViBe	0.3666	0.3617	0.3748
Average	0.2397	0.2226	0.2438

**Table 18:** F-m_s_ of the evaluated BS algorithms tested on Sequence_2.

	BS	BS + M	BS + MM
AdaptiveMedian	0.0317	0.0387	0.0544
Bayes	0.1683	0.0911	0.0105
Codebook	**0.1865**	**0.2771**	**0.3714**
Gaussian	0.0964	0.1109	0.1435
GMG	0.0709	0.1477	0.1931
GMM1	0.0572	0.0565	0.0573
GMM2	0.0001	0	0
GMM3	0.0421	0.0435	0.0470
KDE	0.0041	0.0015	0
KNN	0.1114	0.0328	0.0027
PBAS	**0.2665**	**0.3244**	**0.3450**
PCAWS	0	0	0
Sigma-Delta	0.1771	0.1854	0.1860
SOBS	**0.2182**	**0.2850**	**0.3274**
Texture	0.1574	0.1543	0.1008
ViBe	0.0520	0.0677	0.0899

**Table 19:** Average F-m_s_ of the evaluated BS algorithms test on Sequence_5 and Sequence_6.

	BS	BS + M	BS + MM
Sequence_5	0.0773	0.0842	0.0631
Sequence_6	0.0297	0.0313	0.0175

**Table 20:** Average F-m_s_ of Sequence_5 and Sequence_6 detected by the evaluated BS algorithms.

	BS	BS + M
AdaptiveMedian	0.0265	0.0011
Bayes	**0.1036**	**0.1366**
Codebook	0.0345	**0.1412**
Gaussian	**0.1130**	0.1054
GMG	0.0091	0.0312
GMM1	0.0460	**0.1108**
GMM2	0.0001	0
GMM3	0.0128	0.0374
KDE	0.0097	0
KNN	**0.1193**	0.0571
PBAS	0.0708	**0.1313**
PCAWS	0	0
Sigma-Delta	**0.1712**	0.1086
SOBS	0.1018	0.0592
Texture	0.0008	0.0010
ViBe	0.0367	0.0036

**Table 21:** Computational load and memory usage of the evaluated BS algorithms.

BS	Memory Usage		Computational Load	
	USS (kb)	RSS (kb)	Execution Time (ms/Frame)	CPU Occupancy ^1^ (%)
Adaptive Median	**15,844**	**24,760**	11.65	58.41
GMG	131,524	140,432	16.58	68.89
Gaussian	**19,352**	**28,172**	21.44	79.07
GMM1	30,060	39,100	27.59	81.03
GMM2	34,292	43,216	38.02	87.55
GMM3	27,680	36,540	31.99	80.66
Codebook	102,640	111,328	**6.09**	**24.18**
Bayes	307,752	316,672	123.31	95.45
KDE	51,896	60,844	**10.33**	**54.36**
KNN	195,972	204,788	39.29	84.39
PBAS	103,336	112,332	345.73	97.39
PCAWS	422,696	431,596	594.84	98.55
Sigma-Delta	**16,336**	**25,328**	15.63	70.27
SOBS	74,008	82,824	223.29	97.13
Texture	132,192	14,1048	3157.05	99.64
ViBe	23,680	32,672	**7.66**	**44.07**

^1^ In this experiment, CPU occupancy is the percentage based on one core. For this computer with eight cores, the maximum CPU occupancy is 800%.

**Table 22:** The Evaluation Results of the BS, and BS with post-processing with the BGSLibrary.

BS		BS		BS + M			BS + MM		
	Pr_d_	Re_d_	F-m_d_	Pr_d_	Re_d_	F-m_d_	Pr_d_	Re_d_	F-m_d_
AdaptiveBGLearning	0.556	0.267	0.361	0.551	0.378	0.448	0.535	0.488	0.511
AdaptiveSelectiveBGLearning	0.604	0.162	0.256	0.597	0.213	0.313	0.571	0.268	0.364
FrameDifference	0.213	0.421	0.283	0.17	0.601	0.265	0.11	0.641	0.188
FuzzyAdaptiveSOM [64]	0.208	0.323	0.253	0.202	0.485	0.285	0.193	0.649	0.298
FuzzyChoquetIntegral [47]	0.114	0.178	0.139	0.104	0.191	0.134	0.081	0.197	0.115
FuzzyGaussian [11,73]	0.701	0.05	0.094	0.704	0.074	0.135	0.679	0.092	0.162
FuzzySugenoIntegral [74]	0.089	0.226	0.128	0.081	0.276	0.125	0.058	0.306	0.097
GMM-Laurence [40]	0.596	0.163	0.256	0.589	0.218	0.318	0.563	0.277	0.371
LOBSTER [48]	0.206	0.983	0.34	0.204	0.985	0.337	0.202	0.99	0.335
MeanBGS	0.051	0.695	0.096	0.033	0.644	0.063	0.019	0.536	0.037
MultiLayer [75]	0.362	0.762	0.491	0.357	0.769	0.488	0.35	0.779	0.483
PratiMediod [55,76]	0.299	0.874	0.445	0.293	0.896	0.442	0.286	0.919	0.436
SimpleGaussian [28]	0.717	0.041	0.078	0.722	0.064	0.118	0.7	0.08	0.144
StaticFrameDifference	0.626	0.082	0.145	0.621	0.111	0.188	0.593	0.134	0.219
SuBSENSE [68]	0.175	0.989	0.297	0.174	0.99	0.295	0.172	0.991	0.294
T2FGMM_UM [77]	0.088	0.995	0.161	0.077	0.999	0.143	0.073	0.999	0.136
T2FGMM_UV [77]	0.605	0.188	0.286	0.596	0.32	0.417	0.566	0.449	0.501
T2FMRF_UM [78]	0.058	0.968	0.11	0.047	0.986	0.091	0.039	0.999	0.075
T2FMRF_UV [78]	0.35	0.534	0.423	0.343	0.743	0.469	0.323	0.855	0.469
Texture2 [45] ^2^	0.397	0.344	0.369	0.39	0.376	0.383	0.381	0.406	0.393
TextureMRF [46]	0.356	0.149	0.21	0.346	0.167	0.225	0.335	0.185	0.238
VuMeter [79]	0.722	0.025	0.048	0.735	0.055	0.103	0.714	0.089	0.158
WeightedMovingMean	0.107	0.677	0.185	0.078	0.705	0.141	0.045	0.644	0.083
WeightedMovingVariance	0.136	0.624	0.223	0.109	0.662	0.188	0.076	0.63	0.136

^2^ The implementation of Texture in the BGSLibrary [1] is different from the description in the original paper [45], so the evaluation result shown in Table 22 is also different from the result of the implementation in the previous experiments shown in Table 9. To distinguish these two implementations, we name the implementation in BGSLibrary as Texure2.

## Supplementary Materials

Remote Scene IR Dataset and the foreground masks detected by each evaluated BS algorithm are available online: https://github.com/JerryYaoGl/BSEvaluationRemoteSceneIR.
